# Transarterial chemoembolization plus apatinib with or without camrelizumab for unresected hepatocellular carcinoma: A two-center propensity score matching study

**DOI:** 10.3389/fonc.2022.1057560

**Published:** 2022-11-10

**Authors:** Di Zhu, Kun Ma, Wei Yang, Hai-Feng Zhou, Qi Shi, Jian-Wu Ren, Yu-Guan Xie, Sheng Liu, Hai-Bin Shi, Wei-Zhong Zhou

**Affiliations:** ^1^ Department of Interventional Radiology, The First Affiliated Hospital of Nanjing Medical University, Nanjing, China; ^2^ Department of Interventional Radiology, The Affiliated Hospital of Nanjing University of Chinese Medicine, Nanjing, China

**Keywords:** apatinib, immunotherapy, transarterial chemoembolization, hepatocellular carcinoma, PD-1, prognosis

## Abstract

**Purpose:**

To compare the effectiveness and safety of transarterial chemoembolization (TACE) combined with apatinib and camrelizumab with those of TACE as well as apatinib among patients with unresectable hepatocellular carcinoma (HCC).

**Materials and methods:**

The data of patients with unresectable HCC (uHCC) who received TACE-apatinib-camrelizumab combination (TACE + AC group) and TACE-apatinib combination (TACE + A group) were collected from two centers between January 2018 and January 2022. Propensity score matching (PSM) was conducted to diminish the bias between the two groups. The primary outcome measures of the study were overall survival (OS) and progression-free survival (PFS), and the secondary outcome measures were response rate (ORR), disease control rate (DCR), and adverse events (AEs).

**Results:**

A total of 102 patients were enrolled in this study after PSM, with 34 patients in the TACE + AC group and 68 patients in the TACE + A group. Compared to the TACE + A group, TACE + AC had a significantly longer median OS (25.5 months, interquartile range [IQR], 23.5–33.0) than 18.5 months (IQR, 13.0–25.0; *P* = 0.001). Similarly, the PFS of the TACE + AC group was significantly improved (14.0 months, IQR, 9.0–NA) compared to that of the TACE + A group (5.0 months, IQR, 2.5–9.0; P = 0.001). The ORR rates (55.9% vs. 51.5%), and DCR rates (79.4% vs. 72.1%) were comparable between groups (*P* > 0.05). All treatment-related adverse events were tolerable and manageable, and no serious adverse events were observed.

**Conclusion:**

TACE combined with apatinib plus camrelizumab demonstrated superior efficacy to TACE plus apatinib for patients with unresectable HCC. The two combination therapies showed similar safety profiles.

## Introduction

Hepatocellular carcinoma (HCC) is the second leading cause of cancer-related deaths worldwide and is often diagnosed at an advanced stage because of its insidious onset and nonspecific symptoms. Transarterial chemoembolization (TACE) and systematic therapy are considered standard therapeutic methods for patients with intermediate and advanced HCC, respectively (1–3). As a widely accepted and proven treatment strategy for HCC, TACE could effectively inhibit tumor progression. However, TACE could cause hypoxia in tumor tissue, which ultimately induces the expression of vascular endothelial growth factor (VEGF) and increases tumor angiogenesis (4), and consequently, mediates tumor growth and/or metastasis. Moreover, repeated TACE procedures can gradually impair liver function and aggravate liver cirrhosis.

Apatinib (Jiangsu Hengrui Medicine Co., Ltd., Jiangsu, China), a novel targeted agent, has higher selectivity to VEGFR-2 than sorafenib. Qiu et al (5) proposed that TACE combined with apatinib can improve the efficacy of unresectable HCC compared to TACE alone. Meanwhile, camrelizumab (SHR-1210, Jiangsu Hengrui Medicine Co., Ltd., Jiangsu, China) is a humanized monoclonal antibody against PD-1. According to the RESCUE trial (6), camrelizumab in combination with apatinib has an efficacy profile of 34.3% for advanced HCC. Moreover, with the continuation of the IMBrave150 study (7) and several clinical trials (8, 9), immunotherapy in combination with antiangiogenic drugs is known to significantly improve the outcome of patients with advanced HCC. Furthermore, evidence (10) shows that TACE is an inducer of immunogenic cell death, resulting in facilitating antigen presentation and priming of antitumour lymphocytes (11). Thus, there is an appealing rationale for the combination of TACE, tyrosine kinase inhibitors (TKIs), and immune checkpoint inhibitors (ICIs) (12).

Several studies (13, 14) have shown that TACE combined with anti-angiogenic therapy and immunotherapy can improve the treatment efficacy of patients with unresectable HCC, with an ORR of approximately 35%–59% and median overall survival (OS) of approximately 13–35 months. Few studies have been conducted using TACE along with apatinib and camrelizumab for patients with unresectable HCC. Therefore, we conducted this retrospective study to determine the efficacy and safety of TACE combined with apatinib and camrelizumab (TACE + AC) therapy compared to TACE combined with apatinib (TACE + A) therapy.

## Materials and methods

### Study design and patient selection

This retrospective analysis was conducted between January 2018 and January 2022, on all patients with unresectable HCC from the First Affiliated Hospital of Nanjing Medical University and the Affiliated Hospital of Nanjing University of Chinese Medicine who received TACE plus apatinib with/without camrelizumab. The study was approved by the Institutional Ethics Review Boards of both hospitals, and the procedures followed in this study were conducted in accordance with the guidelines of the World Medical Association Declaration of Helsinki. The requirement for informed consent was waived due to the retrospective nature of the study. According to the Guidelines for Diagnosis and Treatment of Primary Liver Cancer in China, HCC was diagnosed pathologically or clinically. The inclusion criteria for the study were as follows: (1) Barcelona Clinic Liver Cancer (BCLC) stage B or C; (2) Child–Pugh class A5–B7; (3) Eastern Cooperative Oncology Group (ECOG) performance status ≤ 1; and (4) ≥ 1 cycle of TACE and apatinib with or without camrelizumab. The exclusion criteria were as follows: (1) < 1 month of apatinib or camrelizumab treatment; (2) appearance of secondary primary malignant tumors; (3) contraindication to camrelizumab (an allergy to the active ingredient and excipients of camrelizumab); and (4) incomplete data or loss to follow-up.

### TACE procedure

TACE was initiated before apatinib and camrelizumab administration. Under local anesthetic, TACE treatment was conducted through the femoral artery. To determine the number, size, location, and feeding arteries of the tumors, a 5-F catheter (COOK) was inserted and angiography was performed. Then, an emulsion of chemotherapeutic drugs (lobaplatin, 30–50 mg; epirubicin, 10–30 mg) mixed with lipiodol was administered through the hepatic artery. Thereafter, embolization *via* a microcatheter (2.7 F; Terumo Medical Corp., Tokyo, Japan; or 2.4 F; Merit Maestro, South Jordan, Utah, USA) was performed either selectively or superselectively. Selective embolization with 300 μm polyvinyl alcohol particles (Biosphere Medical, Paris, France; or Jiangsu Hengrui Medicine Co., Ltd., Jiangsu, China) or gelatin sponge particles was performed to achieve blood flow stasis in the tumor-feeding artery. Post-TACE syndrome was recorded, and liver function indices were assessed within 1 week of each TACE session.

### Apatinib and camrelizumab administration

Apatinib was administered orally 250 mg once a day within 1 week of the initial TACE and was suspended 3 days before and after repeated TACE procedures. Camrelizumab was administered 200 mg intravenously within 1 week of the initial TACE and then every 3 weeks continuously (maximum of 24 months of camrelizumab treatment). The doses of camrelizumab and apatinib were reduced, suspended, or discontinued in patients who experienced severe adverse events (AEs).

### Follow-up

All patients were followed up constantly until death or the end of the study (March 1, 2022). To track treatment-related adverse events (AEs), blood tests, including complete blood counts, liver, kidney, cardiac biomarkers, and thyroid functions, were conducted approximately every 3 weeks. Tumor markers and contrast-enhanced computed tomography (CT) or magnetic resonance imaging (MRI) were performed every 2 months to assess the treatment response. TACE was repeated in keeping with the tumor status, liver function, and patient’s general condition when residual viable tumors were detected or new lesions emerged after a multidisciplinary team discussion.

### Assessment and outcomes

The primary measure outcomes were OS and progress-free survival (PFS). OS was defined from the date of the first TACE therapy to the date of death arising from any cause or the date of the last contact in both groups. PFS was defined as the time between the beginning of TACE treatment and the first sign of tumor progression or death. Secondary measure outcomes of this study included the objective response rate (ORR), disease control rate (DCR), and AEs. The tumor response was evaluated by two experienced radiologists using the modified Response Evaluation Criteria in Solid Tumors (mRECIST, version 1.1), including complete response (CR), partial response (PR), stable disease (SD), and progressive disease (PD). The ORR was defined as CR + PR, and the DCR was defined as CR + PR + SD. AEs were assessed based on the Common Terminology Criteria for Adverse Events (CTCAE, version 4.03).

### Statistical analyses

Propensity score matching (PSM) was performed to minimize the effects of selection bias and potential confounders. Categorical data are expressed as the number of patients (percentage). Quantitative data were expressed as mean ± standard deviation and median (range) for normally and nonnormally distributed variables. Categorical data between the two groups were compared using the c2 test or Fisher’s exact test, as appropriate. Quantitative data were compared using Student’s t-test or Mann–Whitney U test, as appropriate. Survival curves were analyzed by Kaplan–Meier method using the log-rank test. All statistical analyses were performed using SPSS Statistics version 26 (IBM, Armonk, New York, USA). All statistical tests were two-tailed, and *P* < 0.05 was considered statistically significant.

## Results

### Patient demographics

**Figure 1:**
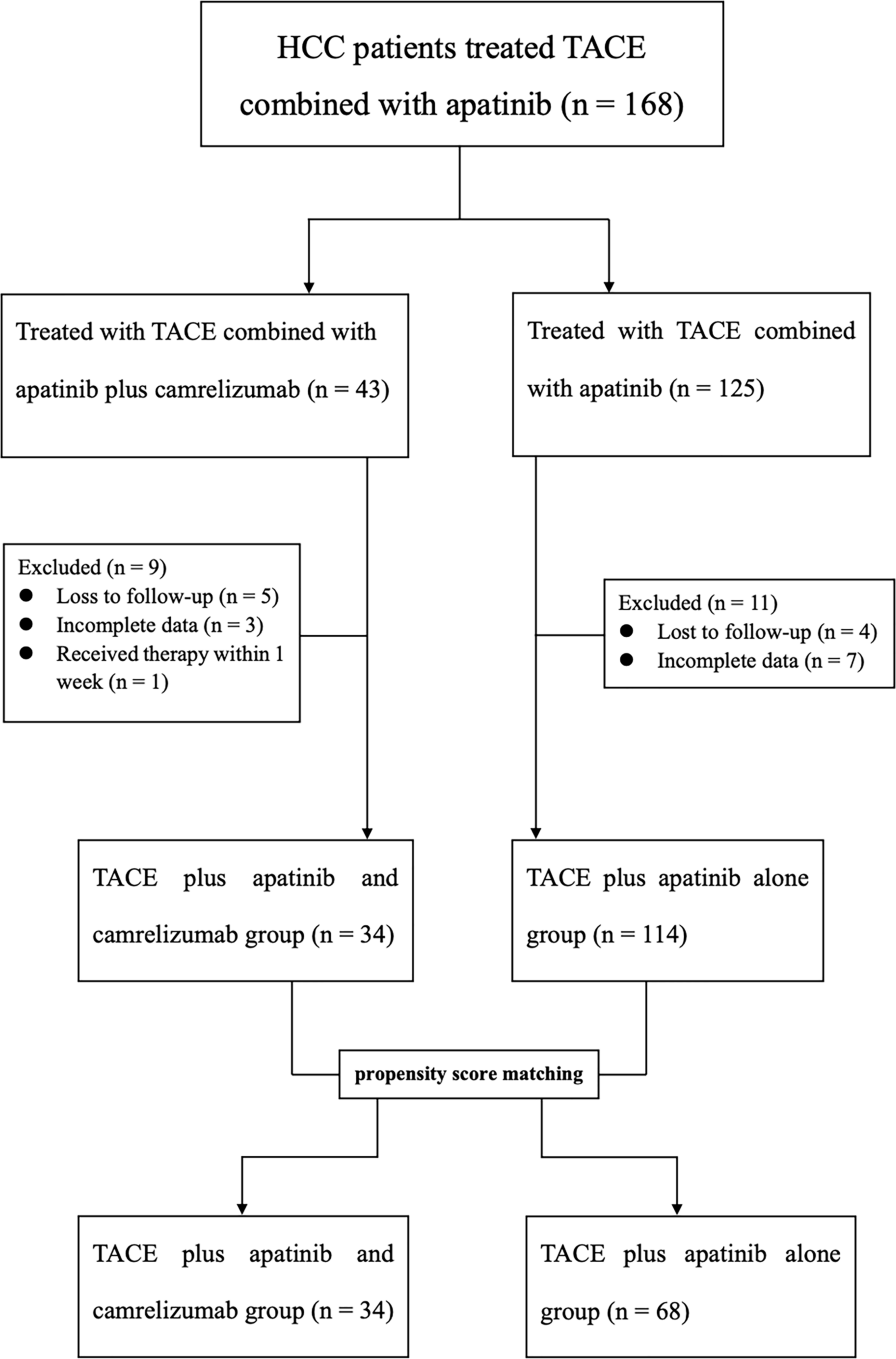
Flow diagram of patient enrollment. HCC, Hepatocellular carcinoma; TACE, Transarterial chemoembolization.

Between January 2018 and January 2022, 147 patients with BCLC stage B or C were considered eligible for this study, including the TACE + AC group (n = 34) and TACE + A group (n = 113). The median follow-up time is 22.8 months in the TACE + AC group, while 29.3 months in the TACE + A group. The flow diagram is displayed in **Figure 1**. The baseline characteristics of the patients are summarized in **Table 1**. Before PSM, BCLC stage (B/C, *P* = 0.003) and tumor distribution (single/multiple, *P* = 0.02) showed statistically significant differences in the two groups. Groups were matched strictly in age, gender and grade of BCLC classification (caliper = 0.1). After PSM at a 1:2 radio, there were no statistically significant differences in the baseline characteristics between the two groups. A total of 102 patients were included after PSM, among whom, 34 were in the TACE + AC group and 68 were in the TACE + A group.

**Table 1:** Patient characteristics at baseline.

		Before PSM			After PSM		
Characteristic		TACE+AC	TACE+A	p	TACE+AC	TACE+A	p
**Age (years)**				0.32			0.52
	< 60	23	65		23	41	
	≥ 60	11	48		11	27	
**Sex**				0.25			1
	Male	29	84		29	58	
	Female	5	29		5	10	
**ECOG PS**				1			0.67
	0	19	62		19	34	
	1	15	51		15	34	
**Etiology**				0.47			0.78
	HBV	29	89		29	56	
	Other	5	24		5	12	
**Child–Pugh class**				0.79			0.57
	A	30	105		30	56	
	B	4	18		4	12	
**AFP**				0.84			0.40
	< 200 ng/ml	21	67		21	35	
	≥ 200 ng/ml	13	46		13	33	
**Tumor distribution**				0.02			0.55
	Single	12	14		12	22	
	Multiple	22	99		22	46	
**Tumor size**				0.82			0.35
	< 10 cm	27	85		27	47	
	≥ 10 cm	7	28		7	21	
**Extrahepatic metastases**				0.07			0.51
	Yes	13	24		13	21	
	No	21	89		21	47	
**Macrovascular invasion**				0.01			0.2
	Yes	17	17		17	24	
	No	17	96		17	44	
**BCLC stage**				0.00			1
	B	13	73		13	26	
	C	21	40		21	42	

Data are presented as the median (range) or N (%). PSM: Propensity score matching, TACE, Transcatheter arterial chemoembolization; TACE + A, TACE plus apatinib; TACE + AC, TACE plus apatinib and camrelizumab; ECOG PS, Eastern Cooperative Oncology Group Performance Status; BCLC, Barcelona Clinic Liver Cancer; AFP, Alpha-fetoprotein.

### Efficacy

**Figure 2:**
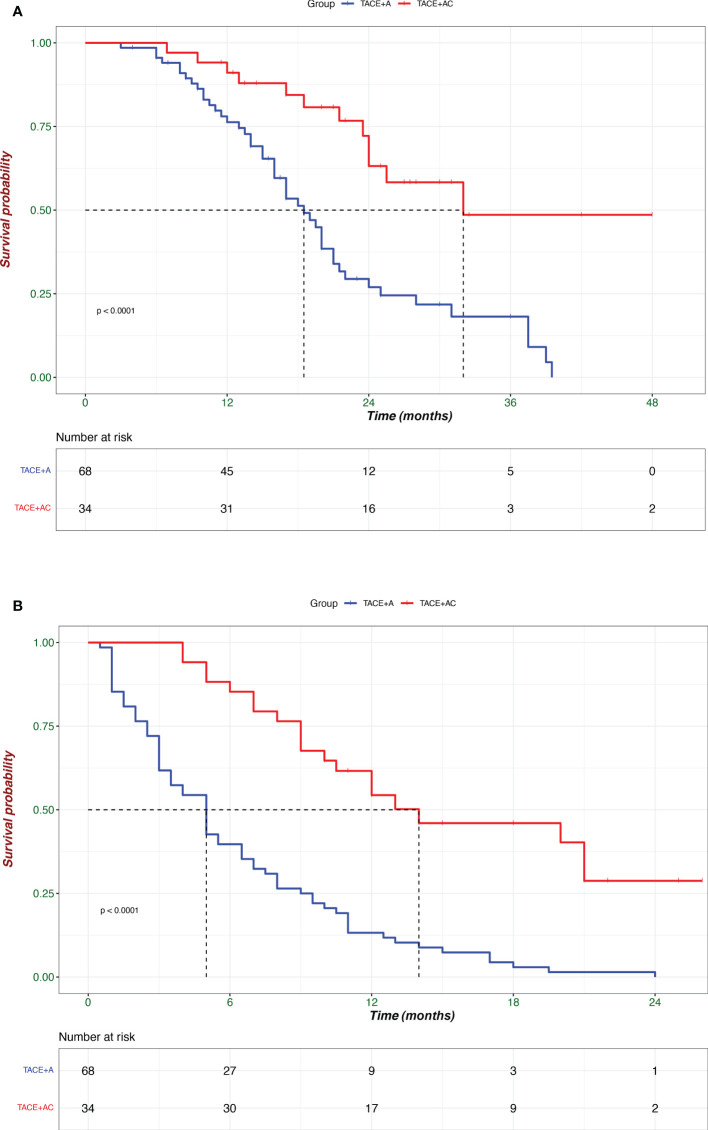
Kaplan–Meier analyses of overall survival **(A)** and progression-free survival **(B)** according to treatment groups. TACE + A, Transarterial chemoembolization combined with apatinib; TACE + AC, Transarterial chemoembolization combined with apatinib plus camrelizumab.

**Figure 3:**
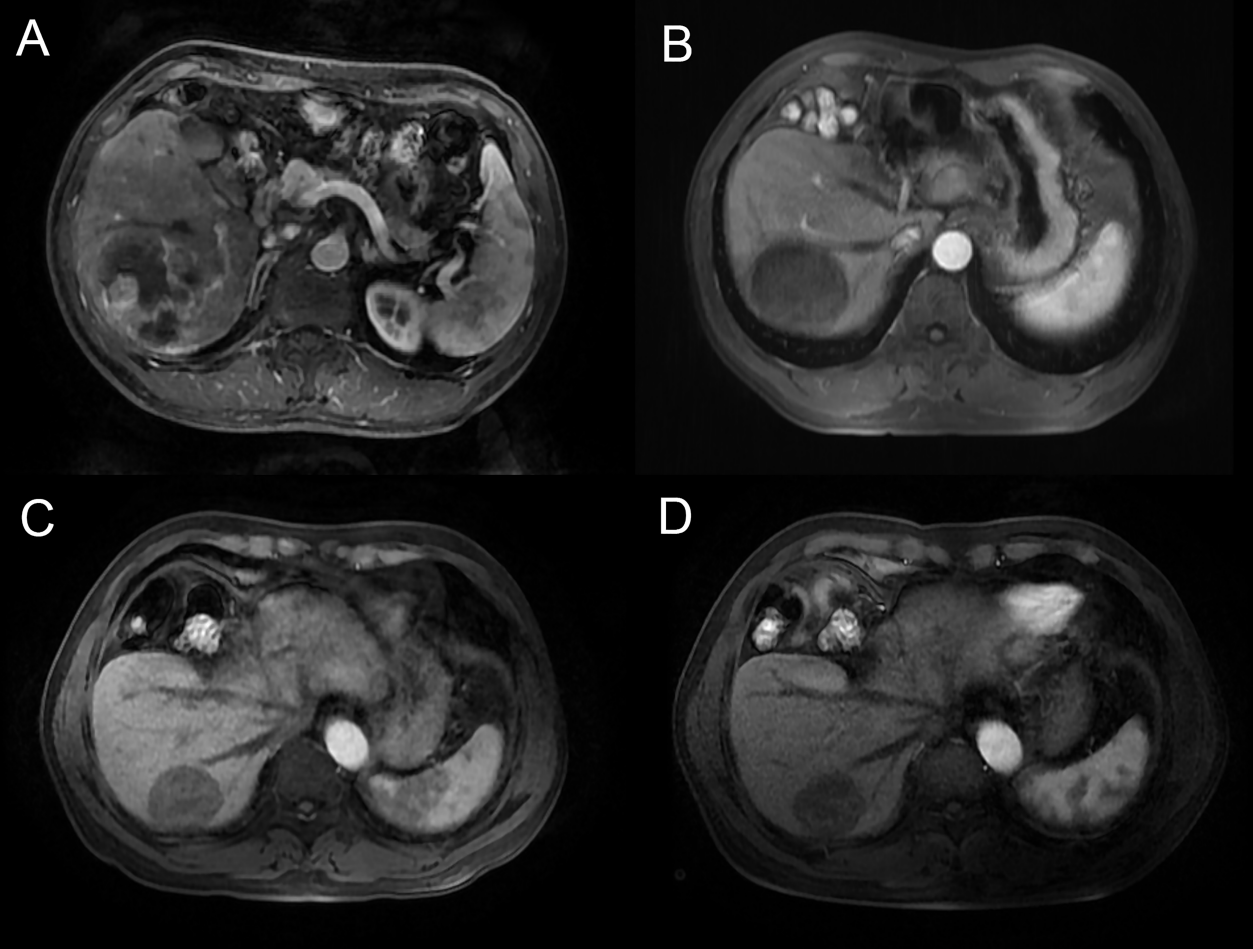
Male, 58y, BCLC B stage, Child-Pugh grade A6, massive and ruptured HCC. MR images (arterial phase) were acquired. **(A)** before TACE treatment, **(B)** 6 months after the combination treatment, **(C)** 12 months after the combination treatment, **(D)** 18 months after the combination treatment, demonstrating complete response, with a reduction in tumor size, the patient was classified as CR according to mRECIST. TACE, transarterial chemoembolization.

Before PSM, Patients in the TACE + AC group had a median OS of 25.5 (IQR: 23.5–33) months and a median PFS of 14.0 (IQR: 9.0–NA) months, while 19.1 (IQR: 2.5–27.5) months and 5.1 (IQR: 2.7-8.1) for those in the TACE + A group, respectively. Patients in the TACE + AC group had a median OS of 25.5 (IQR: 23.5–33) months compared to 18.5 (IQR: 13.0–25.0) months for those in the TACE + A group (HR = 0.312, 95%CI = 0.162–0.602; **Figure 2**); and a median PFS of 14.0 (IQR: 9.0–NA) months compared to 5.0 (IQR: 2.5–9.0) months for those in the TACE + A group. The survival rates of the TACE + AC group were 91.1%, 63.2%, and 48.6% at 1, 2, and 3 years, while those of the TACE + A group were 76.3%, 27%, and 18.2%, respectively. **Figure 3** is the representative MR imaging figures from 1 case of CR. All data and results are available in **Supplementary Tables 1**, **2**.

### Tumor responses

The tumor responses at the 1-year rate of the two groups of patients are shown in **Table 2**. For the TACE + AC group, 4 (11.8%) patients achieved CR, 15 (44.1%) achieved PR, 8 (23.5%) patients were in the SD state, and 7 (20.6%) patients had PD. However, in the TACE + A group, 4 (5.9%) patients achieved CR, 31 (45.6%) patients achieved PR, 14 (20.6%) patients were in the SD state, and 19 (27.9%) had PD. The ORR rates (55.9% vs. 51.5%) and DCR rates (79.4% vs. 72.1%) of the TACE + AC group were numerically higher than those of the TACE + A group, and neither showed a statistically significant difference (*P* > 0.05).

**Table 2:** Tumor response at 1 year between the two groups based on mRECIST1.1.

	TACE+AC group	TACE +A group	X^2^	P-value	Overall
	(n = 34, %)	(n = 68, %)			(n = 102,%)
Tumor response					
CR	4 (11.8)	4 (5.9)	0.99	0.44	13 (12.6)
PR	15 (44.1)	31 (45.6)	0.20	1.00	41 (40.2)
SD	8 (23.5)	14 (20.6)	0.12	0.80	22 (21.6)
PD	7 (20.6)	19 (27.9)	0.71	0.47	26 (25.5)
ORR (CR+PR)	19 (55.9)	35 (51.5)	0.18	0.84	54 (52.9)
DCR (CR+PR+SD)	27 (79.4)	49 (72.1)	0.64	0.48	76 (74.5)

mRECIST, Modified Response Evaluation Criteria in Solid Tumors; CR, Complete response; PR, Partial response; SD, Stable disease; PD, Progressive disease; ORR, Objective response rate; DCR, Disease control rate.

### Safety

No treatment-related deaths were observed and the treatment-related adverse events (TrAEs) are listed in **Table 3**. All toxicities were manageable. AEs of any grade during the TACE procedure included abdominal pain (65.1%), transaminitis (46.1%), fever (53.9%), lymphopenia (10.8%), decreased appetite (29.4%), nausea/vomiting (58.5%), diarrhea (25.5%), fatigue (13.7%), leukopenia (14.7%), neutropenia (11.8%), and anemia (13.7%). There were no significant differences in AEs resulting from TACE between the groups. In contrast, hand-foot syndrome (29.4%), hypertension (44.1%), and reactive cutaneous capillary endothelial proliferation (RCCEP) (23.5%) were the most common AEs in the period of apatinib and camrelizumab administration. In the TACE + AC group, apatinib administration was suspended in five patients and camrelizumab administration was suspended in one patient. In the TACE + A group, apatinib administration was suspended in 10 patients due to intolerance. Grade 4 myelosuppression occurred in one patient after the TACE procedure and recovered after symptomatic management.

**Table 3:** Treatment-related adverse events in the two groups.

		TACE+AC (n = 34)				TACE+A (n = 68)				*P* value
**Adverse events**										
	Toxicity grade	1/2		3/4		1/2		3/4		
**TACE-related**										
	Diarrhea	6	17.6%	1	2.9%	14	20.6%	5	7.4%	0.268
	Transaminitis	13	38.2%	3	8.8%	26	38.2%	5	7.4%	1
	Rash	4	63.7%	0	0.0%	11	16.1%	3	4.4%	0.409
	Nausea with/without vomiting	22	64.7%	2	5.9%	31	45.6%	5	7.4%	0.135
	Abdominal pain	19	55.9%	4	11.7%	42	61.7%	9	13.2%	0.641
	Fatigue	7	29.0%	0	0.0%	4	5.8%	3	4.4%	0.221
	Fever	19	26.5%	3	8.8%	33	48.5%	0	0.0%	0.144
	Leukopenia	6	17.6%	1	2.9%	8	11.7%	0		0.249
	Neutropenia	5	14.7%	1	2.9%	6	8.8%	0		0.208
	Lymphopenia	4	11.8%	1	2.9%	5	7.4%	1	1.5%	0.499
	Thrombopenia	7	20.6%	0	0.0%	12	17.6%	0	0.0%	0.789
	Anemia	6	17.6%	0	0.0%	8	11.7%	0	0.0%	0.543
	Decreased appetite	6	17.6%	0	0.0%	24	35.2%	0	0.0%	0.071
**Apatinib and Camrelizumab -related**	Hand-foot skin reactions	7	11.7%	3	8.8%	20	29.4%	8	11.7%	0.283
	Hypertension	11	32.4%	4	11.7%	16	23.5%	11	16.1%	0.676
	REECP	7	29.0%	1	2.9%	0		0		–
	all	34	100.0%	11	32.3%	64	100.0%	16	23.5%	0.298

## Discussion

Our study revealed that TACE + AC therapy was more effective than TACE + A therapy in patients with unresectable HCC. Patients who received the TACE + AC modality had a median OS of 25.5 (IQR: 23.5–33.0) months and a median PFS of 18.5 (IQR: 13.0–25.0) months, which yielded a sufficient edge over the TACE + A modality and was comparable to the results of previous studies (15, 16).

The combination of TACE with immunotherapy modalities has shown promising clinical efficacy. Regarding the survival time, several retrospective studies have shown that TACE combined with TKIs and ICIs demonstrated superior OS (18–24 months) and PFS (5.5–13.3 months) than TACE combined with TKIs or TKIs combined with ICIs, which were not better than our outcomes. In our study, the median OS and PFS in the TACE + AC group were numerically higher than those in the TACE + A group. Of note, Ju et al. (17, 18) reported comparable results of TACE combined with apatinib and camrelizumab; thus, our findings demonstrated a substantial and synergistic improvement in survival for patients with unresectable HCC treated with TACE + AC. Several possible explanations exist for this finding (1) TACE can induce the up-regulation of VEGF and neovascularization, and apatinib can inhibit tumor angiogenesis by targeting VEGFR-2 (12); (2) TACE can cause tumor cell necrosis and neoangiogenesis, while the immune tolerance induced by TACE can be attenuated by TKI and PD-1 inhibitors (19, 20); and (3) the combination of ICIs with TKIs can convert “cold tumors” into “hot tumor” by T cell activation (11), which may restore exhausted T cells and facilitate anti-tumor immunity (21). Therefore, patients with unresectable HCC may experience superior clinical results when applying TACE, apatinib, and camrelizumab in combination.

For the tumor response, the RESCUE trial (6) disclosed an ORR of 34.3% and a DCR of 77.1% following combined apatinib and camrelizumab therapy. The TACE + AC group had a greater ORR compared to those reported by the IMbrave150 trial (7) (atezolizumab plus bevacizumab: ORR = 33.2%), the phase 1b KEYNOTE-524 trial (22) (lenvatinib plus pembrolizumab: ORR = 46.0%), and the ORIENT-32 trial (sintilimab plus bevacizumab: ORR = 24%); this was likely because the addition of TACE is thought to be related to immune activation and can induce low expression of Tregs *via* modulating pro-inflammatory pathways. In our study, the CR, ORR, and DCR rates of the TACE + AC group were numerically greater than those of the TACE + A group (11.8% vs. 5.9%, 55.9% vs. 51.5%, and 79.4% vs. 72.1%, respectively), although the difference did not reach statistical significance. Simultaneously, Ju et al. reported an ORR of 58.8% and a DCR of 81.2% in the TACE + AC group, which is similar to our findings. However, our results did not achieve statistical significance, likely for the following reasons: (1) the intervals between TACE were believed to affect the results, (2) some patients in the TACE + AC group were newly included in the cohort and had comparatively fewer cycles of camrelizumab, and (3) PVTT and subsequent metastasis can induce tumor cells to spread, which may reduce the efficacy of immunotherapy in patients with PVTT. These findings and views were similarly shared by Cai et al. (23).

Regarding AEs, the most common AEs were hand-foot skin reaction and hypertension, which were predominantly related to apatinib. Moreover, the incidence of apatinib-related AEs (grade ≥ 3) was 11%–16%, whereas events such as increased AST/ALT and RCCEP were related to camrelizumab. According to previous studies, the most common AE in patients with HCC treated by TACE is embolization syndrome, including pain, fever, nausea, and vomiting. Altogether, TACE + AC therapy for patients with unresectable HCC presented a safe profile.

This study also has some limitations. First, this is a retrospective study with a small sample of enrolled patients and a short follow-up period. Therefore, the results may not be generalizable and should be interpreted with caution. Second, although we performed PSM to avoid selection bias, our analyses may still be influenced by some inherent biases, such as regional bias and population and tumor-related factors; indeed, the etiology of HCC, prevalence of cirrhosis, comorbidities, and overall treatment approach differs in some regions of the world. Thirdly, the subgroup analyses were lacked in this study. Lastly, some patients did not achieve endpoint events throughout the limited follow-up period.

In conclusion, this study showed that TACE combined with apatinib and camrelizumab therapy demonstrated superior efficacy to TACE combined with apatinib for patients with unresectable HCC. Although promising, our results need to be validated by more studies in the future.

## Data availability statement

The original contributions presented in the study are included in the article/**Supplementary Material**. Further inquiries can be directed to the corresponding author.

## Ethics statement

Written informed consent was obtained from the individual(s) for the publication of any potentially identifiable images or data included in this article.

## Author contributions

Designing and instructing the study: WZZ. Collecting the data: DZ, YGX, JWR and QS. Analyses and interpretation of data: WY and HFZ. Dradting of manuscript: DZ and KM. Critical revision of manuscript: SL, HBS and WZZ. All authors contributed to the article and approved the submitted version. DZ and KM contributed equally this work.

## Funding

This work was supported by the National Natural Science Foundation for Young Scholars of China (81701802).

## Acknowledgments

We thank the nurses (Zhong-ling Pei, Yan Shen, Ling-ling Wu) for doing the follow-up work.

## Conflict of interest

The authors declare that the research was conducted in the absence of any commercial or financial relationships that could be construed as a potential conflict of interest.

## Publisher’s note

All claims expressed in this article are solely those of the authors and do not necessarily represent those of their affiliated organizations, or those of the publisher, the editors and the reviewers. Any product that may be evaluated in this article, or claim that may be made by its manufacturer, is not guaranteed or endorsed by the publisher.
